# A clinical perspective of parathyroid hormone related hypercalcaemia

**DOI:** 10.1007/s11154-019-09529-5

**Published:** 2019-12-03

**Authors:** Chau H. Han, Christopher H. Fry, Pankaj Sharma, Thang S. Han

**Affiliations:** 1 Stanhope Surgery, Stanhope Road, Waltham Cross, EN8 7DJ UK; 2 School of Physiology, Pharmacology and Neuroscience, University of Bristol, Bristol, UK; 3 Institute of Cardiovascular Research, Royal Holloway, University of London, Egham, UK; 4 Department of Endocrinology, Ashford and St Peter’s NHS Foundation Trust, Chertsey, UK

**Keywords:** Ectopic parathyroid adenoma, Familial hypocalciuric hypercalcaemia, Multiple endocrine neoplasm (MEN) syndromes, Calcium/creatinine clearance ratio

## Abstract

There are many causes of hypercalcaemia including hyperparathyroidism, drugs, granulomatous disorders and malignancy. Parathyroid hormone (PTH) related hypercalcaemia is most commonly caused by primary hyperparathyroidism (PHPT) and more rarely by familial hypocalciuric hypercalcaemia (FHH). Algorithms for diagnosis of PTH related hypercalcaemia require assessment of a 24-h urinary calcium and creatinine excretion to calculate calcium/creatinine clearance ratio and radiological investigations including ultrasound scan and ^99m^Tc-sestamibi-SPECT/CT. To illustrate investigations and management of parathyroid-related hypercalcaemia, we present a selection of distinct cases of PHPT due to eutopic and ectopic parathyroid adenomas, as well as a case with a syndromic form of PHPT (multiple endocrine neoplasia type 1), and a case with FHH type 1 due to a *CASR* inactivating mutation. Additional cases with normocalcaemic hyperparathyroidism and secondary hyperparathyroidism are included for completeness of differential diagnosis. The common eutopic parathyroid adenomas are easily treated with parathyroidectomy while the less common ectopic parathyroid adenomas require more complex investigations and operative procedures such as video-assisted thoracoscopic surgery. On the other hand, the much less common FHH does not require treatment. Assessment of kin with FHH is important to identify members with this inherited condition in order to prevent unnecessary interventions.

## Introduction

Calcium (Ca) is essential for a wide variety of body functions, ranging from: bone metabolism, muscle contraction, nerve conduction, hormonal release to blood coagulation. Ca exerts its action on some target organs by activation of a Ca sensing receptor (CaSR), which is primarily located on parathyroid glands and in renal tubules [1]. However, in other tissues, for example in cardiac/smooth muscle Ca^2+^-selective ion channels mediate actions of extracellular Ca. The average adult human body contains approximately 1 kg of Ca of which the majority (99%) is stored in the bone and teeth, while only 1% of the stored Ca (10 g) is readily accessible to physiological processes [2]. The reference range for total plasma Ca is within the range 2.1–2.6 mmol/l, or 8.4–10.4 mg/dl, (this may vary between laboratories), of which the ionised fraction is biologically active and represents about 50% of the total; the remainder is bound to albumin (about one-third), globulins or small anions [3]. Because of variation in albumin levels, correction is usually made to adjust measured total plasma Ca levels to account for this variation in clinical practice, to produce free ionised Ca^2+^ [4]. Under normal circumstances, homeostatic regulation of Ca^2+^ is maintained by parathyroid hormone (PTH), and vitamin D through a negative feedback system which controls plasma Ca^2+^ concentrations, and hence total Ca, to within the reference range. If circulating Ca^2+^ levels start to rise above this range, CaSRs on parathyroid chief cells are activated leading to the inhibition of PTH release. Thus, its actions on tubular reabsorption of Ca^2+^ and formation of the active 1,25-(OH)_2_ vitamin D in the kidney are reduced, thereby restoring plasma Ca^2+^ levels to normal [5, 6].

Hypercalcaemia occurs when there is an override of normal Ca regulation, caused by a number of conditions including hyperparathyroidism, drugs, granulomatous disorders and malignancy. PTH-related hypercalcaemia, a common endocrine condition, is most frequently caused by primary hyperparathyroidism (PHPT) and very rarely by familial hypocalciuric hypercalcaemia (FHH). The annual incidence of PHPT is 3% in the UK [7] and 0.6% in Hong Kong [8], with a prevalence ranging from 1 in 680 in the UK [7], 1 in 2000 in Australia [9] to 1 in 3000 in Hong Kong [8]. There is a female predominance in this condition [10] with a peak prevalence of 1 in 335 in post-menopausal women aged 50–59 years [7, 11]. On the other hand, the prevalence of FHH is estimated to be 1 in 78,000 among the UK population [12].

The present review provides a clinical perspective of PTH-related hypercalcaemia, with a focus on the differentiation of the two contrasting benign conditions of PHPT and FHH. A selection of case studies representative of these conditions is presented to exemplify common, and at times challenging, diagnosis and management.

## Anatomical location of parathyroid adenomas

There are commonly four parathyroid glands that usually are located posterior to the thyroid gland. PTH in patients with PHPT is autonomously secreted by the abnormal parathyroid gland which may arise from hyperplasia, adenoma or carcinoma of the parathyroid glands. Solitary parathyroid adenoma, the most frequent cause of PHPT, is responsible for 85–90% of cases with about 10–15% being double adenomas or multigland hyperplasia [9, 13–15]. The majority of parathyroid adenomas are eutopic while far fewer are ectopic, comprising 9–16% of all cases of hyperparathyroidism [16, 17]. Ectopic parathyroid glands evolve from aberrant migration of the third and fourth brachial pouch during early stages of embryonic development and may occur anywhere along the tract descending from within the thyroid gland to the mediastinum [18]; the most common sites being located in the thymus (30–38%) and retroesophageal region (22–31%), while 18–22% are embedded in the thyroid gland, 22% in the anterosuperior mediastinum and 14% in the posterosuperior mediastinum [14, 16]. Other less common ectopic sites include the thyrothymic ligament, submandibular gland, tracheoesophageal groove and carotid sheath.

## Clinical features and pathophysiology

Although asymptomatic disease is common, patients may develop gastroenterological disorders including constipation, indigestion, nausea and vomiting, acute pancreatitis and peptic ulcers due to increased gastric acid secretion [19]. Neurological and neuropsychiatric disturbances may also develop, such as lethargy, fatigue, weakness and depression [20]. Patients may experience bone pain from osteitis fibrosa cystica which occurs more frequently in areas with high incidence of vitamin D deficiency such as India [21].

Excessive PTH levels lead to hyperstimulation of osteoclasts and diminished activity of osteoblasts resulting in osteopenia/osteoporosis. Hyperparathyroidism also hyperstimulates CaSR and accelerates the rate of vitamin D conversion to its active form (1,25-(OH)_2_ vitamin D), thus patients with PHPT are at greater risk of vitamin D insufficiency than eucalcaemic individuals [22], consequently, Ca accumulates in the kidneys and results in the formation of renal calculi and if left untreated, can lead to kidney injury. However up to a third of PHPT patients have Ca in the reference range [23].

Another important role of PTH is its regulation of plasma phosphate levels. PTH inhibits the reabsorption of phosphate from the proximal tubule [24] while enhances intestinal phosphate absorption mediated by PTH-stimulated vitamin D production [25]. The phosphaturic effect of PTH overwhelms its intestinal action therefore low phosphate levels are often observed in PHPT.

In contrast to PHPT, FHH is an inherited disorder with a strong family history, characterised by lifelong hypercalcaemia with normal (80% of patients) or mildly raised PTH levels [26] and hypocalciuria. The characteristic hypocalciuric hypercalcaemia is explained by the lack of functional CaSRs in FHH whereby the parathyroid gland and kidney fail to detect the levels of circulating Ca [27]. Thus PTH continues to be released despite elevated levels of circulating Ca. Concurrently, the reduced CaSR activity in FHH leads to increased Ca reabsorption in the thick ascending limb of loop of Henle, resulting in hypocalciuria [1]. Patients with FHH are asymptomatic and do not have end-organ damage and parathyroidectomy does not correct hypercalcaemia.

### Genetic basis of PHPT and FHH

Over 10% of patients with PHPT will have a mutation in one of these eleven genes: *MEN1, CASR, AP2S1, GNA11, HRPT2 (CDC73), CDKNB1A, CDKNB1B, CDKNB2B, CDKNB1C, RET, PTH* [28]. Syndromic and non-syndromic forms of PHPT may occur as familial (hereditary) or as sporadic (non-familial) disorders [29].

Less than 5% of patients with PHPT occur as *syndromic forms of PHPT*, belonging to complex familial disorders comprising multiple endocrine neoplasm syndrome types 1 to 4 (MEN1–4), hyperparathyroidism-jaw tumour syndrome and FHH [30], which may be caused by heterozygous germline mutations of the *MEN1* (chromosomal position: 11q13)*, RET* (10q11.2)*, CDKN1B* (12q13) and *HRPT2 (CDC73)* (1q31.2) or *CASR* (3q21.1) gene. The *non-syndromic PHPT* may occur as a familial disorder (familial isolated hyperparathyroidism, FIHPT) or as a sporadic disorder. FIHPT may be due to heterozygous germline mutations of the *MEN1, HRPT2* or *CASR* genes [31].

FHH is a rare familial autosomal dominant condition with a high degree of penetrance [32]. It is caused by loss-of-function mutations; at least 250 different mutations have been discovered to date [33]. Three variants have been identified which are clinically indistinguishable from one another. FHH type 1, the most common form of FHH, is caused by inactivating *CASR* mutations (chromosomal location: 3q21.1) which lead to loss-of-function of the G protein-coupled receptor CaSR [34]. Homozygous mutations of the *CASR* gene are much more critical and can manifest as neonatal severe primary hyperparathyroidism which is usually lethal [35]. FHH type 2 and FHH type 3, also autosomal dominant disorders, are caused by loss-of-function mutations of *GNA11* (chromosomal location: 19p13) which encodes the G-α11 subunit [36] and *AP2S1* (chromosomal location: 19q13.2-q13.3) which encodes the adaptor-protein 2 σ-subunit [37] respectively.

## Diagnosis and management

Algorithms for diagnosis of PTH related hypercalcaemia require assessment of a 24-h urinary Ca and creatinine excretion to calculate the Ca/creatinine clearance ratio (CCCR), expressed as a percentage (%). CCCR is calculated using the equation: $\frac{UCa/ SCa\kern0em }{UCr/ SCr}\times 100\%$***=***$\frac{UCa\times SCr}{SCa\times UCr}\times 100\%$ [23], where *U*_*Ca*_ (mmol/l) and *S*_*Ca*_ (mmol/l) are urine and serum Ca^2+^, *U*_*Cr*_ (mmol/l) and *S*_*Cr*_ (mmol/l) are urine and serum creatinine, respectively. CCCR: <1% suggests FHH and ≥1% suggests PHPT but when CCCR is between 1% and 2%, a fifth of individuals with FHH can overlap with PHPT while 4% of cases with PHPT may be misclassified as FHH [26]. It has been suggested that this false negative is due to hypersecretion of PTH as a result of vitamin D deficiency that often co-exists with PHPT, leading to increased tubular resorption and thus reduced urinary excretion of Ca [38]. It is therefore important to replete vitamin D deficiency during investigations to avoid misclassification. In addition to biochemical assessment, radiological investigations are conducted including ultrasound scan and ^99m^Tc-sestamibi-SPECT/CT to localise the abnormal parathyroid gland [39–41]. Of interest, studies have shown that larger parathyroid adenomas are associated with lower vitamin D levels, which are more likely to be detected by ^99m^Tc-sestamibi-SPECT/CT [42]. Genetic analysis may be necessary to confirm the underlying cause of individuals suspected of FHH or part of familial syndromic and non-syndromic forms of FIHPT. Other investigations are also necessary including ultrasound scan of the kidneys to rule out renal calculi and dual X-ray absorptiometry (DEXA) to assess bone mineral density.

To illustrate investigations leading to the diagnosis and management of PTH related hypercalcaemia, we present a selection of distinct cases of hypercalcaemia arising from eutopic and ectopic parathyroid adenomas, as well as a case with a syndromic form of PHPT, and a case with FHH type 1 due to a *CASR* inactivating mutation. Additional cases with normocalcaemic hyperparathyroidism and secondary hyperparathyroidism are included for completeness of differential diagnosis.

## Case 1: PHPT due to eutopic parathyroid adenoma

**Fig. 1:**
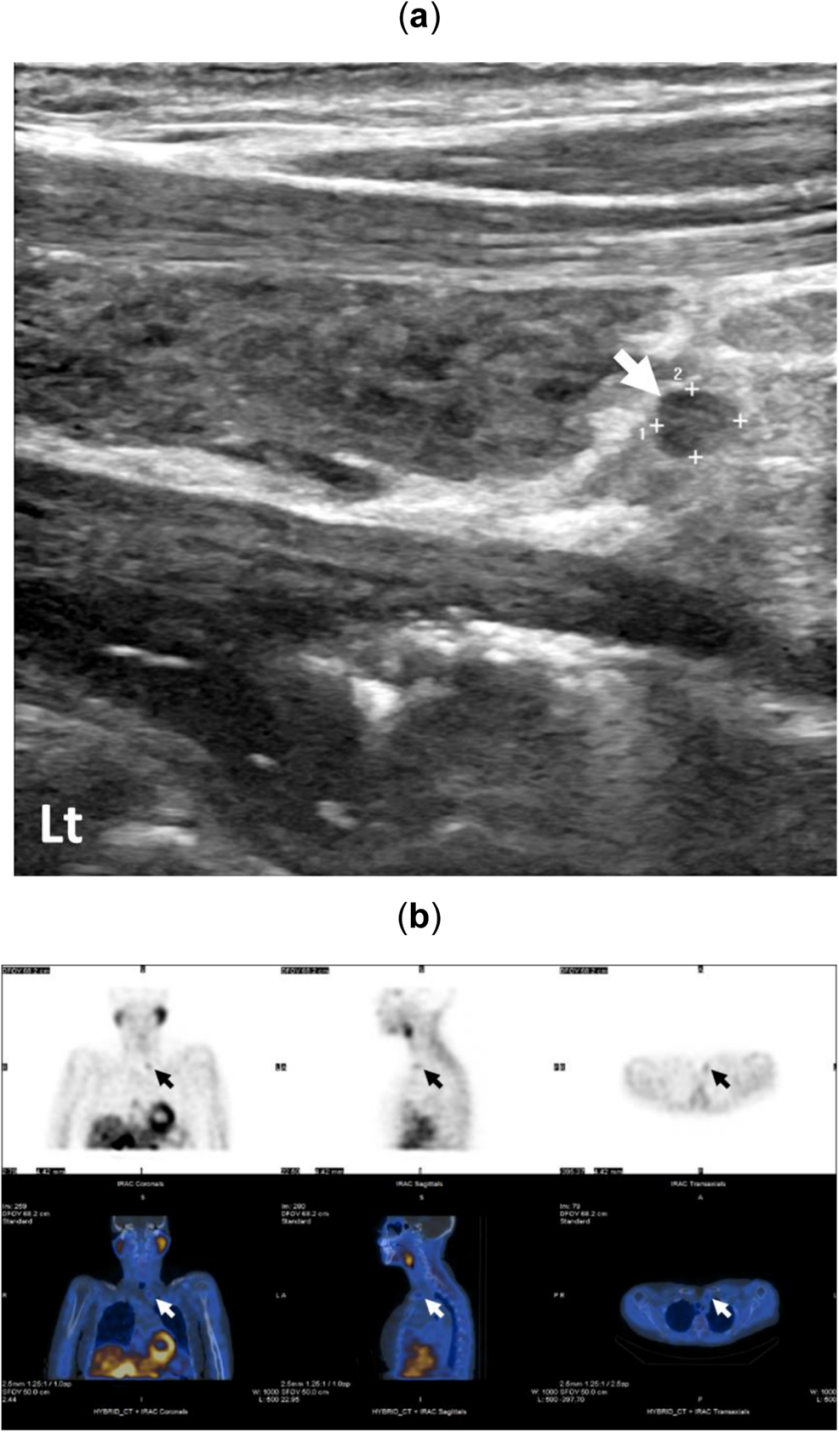
Parathyroid adenoma identified by ultrasound scan measuring 2.6 × 2.1 mm at the inferior aspect of the left thyroid lobe (**a**). Anterior maximum intensity projection images of SPECT showing delayed washout of radiotracer (**b**) and delayed-phase coronal, sagittal and axial fused ^99m^Tc-Sestambi-SPECT/CT images showing a parathyroid adenoma localising posterior to the inferior pole of the left lobe of thyroid (**c**) of a woman (case 1)

A woman in her seventies presented with hypercalcaemia (Ca = 2.80 mmol/l, 11.2 mg/dl) and hyperparathyroidism (PTH = 12.8 pmol/l), and her CCCR was elevated at 1.5% (Table 1) suggesting PHPT. She had vitamin D insufficiency (39 nmol/l) and mild renal impairment (eGFR = 50 ml/min). She had evidence of osteoporosis but without renal calculi. Ultrasound scan and ^99m^Tc-sestamibi-SPECT/CT both showed a eutopic parathyroid adenoma located posterior to the inferior pole of left thyroid lobe (Fig. 1). The patient underwent successful parathyroidectomy by an endocrine surgeon. Post-operative Ca (2.25 mmol/l, 9.0 mg/dl) and PTH (7.2 pmol/l) levels were normalised.

**Table 1:** Clinical characteristics, modes of investigations and treatment of patients with hypercalcaemia

Investigations	Primary hyperparathyroidism					FHH
	Case 1: Eutopic parathyroid adenoma	Case 2: Eutopic parathyroid adenoma	Case 3: Eutopic parathyroid adenoma	Case 4: Ectopic parathyroid adenoma	Case 5: Syndromic PHPT	Case 6: *CASR* inactivating mutation
Age (years)	72	43	75	68	39	43
Family history of hypercalcaemia	None	None	None	None	Under investigation	Mother and maternal grandmother^*^
Calcium (mmol/l, mg/dl)	2.80, 11.2	2.45, 9.8	3.00, 12.0	3.23, 12.9	3.23, 12.9	2.75, 11.0
PTH (pmol/l)	12.8	10.2	10.7	42.1	37.6	4.4
Vitamin D (nmol/l)	39	78	69	57	44	75
Phosphate (mmol/l)	1.01	0.95	0.85	0.87	0.39	0.52
ALP (IU/l)	116	54	92	80	71	18
Creatinine (mol/l)	98	58	58	72	89	61
GFR (ml/min)	50	>60	>60	>60	>90	>90
CCCR (%)	1.5	1.39	2.5	2.8	1.02	0.31
US (or CT) kidneys	No renal calculi	Renal calculi (on CT)	No renal calculi	No renal calculi	Renal calculi (on CT)	No renal calculi
Lumbar spine BMD (T-score)	−2.3	Z-score: −0.7	−1.6	−1.4	Z-score: −0.7	Z-score: +0.2
Femoral BMD (T-score)	−2.7	Z-score: −0.3	−2.6	−2.5	Z-score: −0.6	Z-score: −1.3
Forearm BMD (T-score)	−1.9	Z-score: −0.1	−1.9	−3.8	Z-score: −0.5	–
US neck	Left inferior parathyroid adenoma	Left inferior parathyroid adenoma	No parathyroid adenoma identifiable^†^	No parathyroid adenoma identifiable	Right inferior double parathyroid adenomas	Not required
^99m^Tc-sestamibi-SPECT/CT	Left inferior parathyroid adenoma	Left inferior parathyroid adenoma	No parathyroid adenoma identifiable^†^	Mediastinal ectopic parathyroid adenoma	Right inferior parathyroid adenoma	Not required
Genetic analysis	–	–		–	*MEN1*	*CASR* frameshift variant^‡^
Management	Left inferior parathyroidectomy	Awaiting parathyroidectomy	Right superior parathyroidectomy initially followed by left inferior parathyroidectomy	Left VATS thoracoscopic excision of parathyroid gland	Parathyroidectomy of double adenomas Referred to MEN1 clinic	Family genetic assessment

^*^Patient’s mother and maternal grandmother underwent investigations for calcium without treatment (no genetic analysis performed). ^†^Right parathyroidectomy during initial surgical neck exploration followed by 4D CT scan which revealed parathyroid adenoma in inferior pole of the left lobe. ^‡^See text for details. References ranges: Calcium = 2.1–2.6 mmol/l (8.4–10.4 mg/dl), PTH <8 pmol/l, Vitamin D 75–200 nmol/l, Phosphate 0.8–1.5 mmol/l, ALP 30–130 IU/l, Creatinine 40–90 mol/l, GFR >60 ml/min, CCCR FHH <1%, PHPT >1%, BMI <−1.5 = osteopenia, <−2.5 = osteoporosis

## Case 2: PHPT with normocalcaemic hyperparathyroidism

**Fig. 2:**
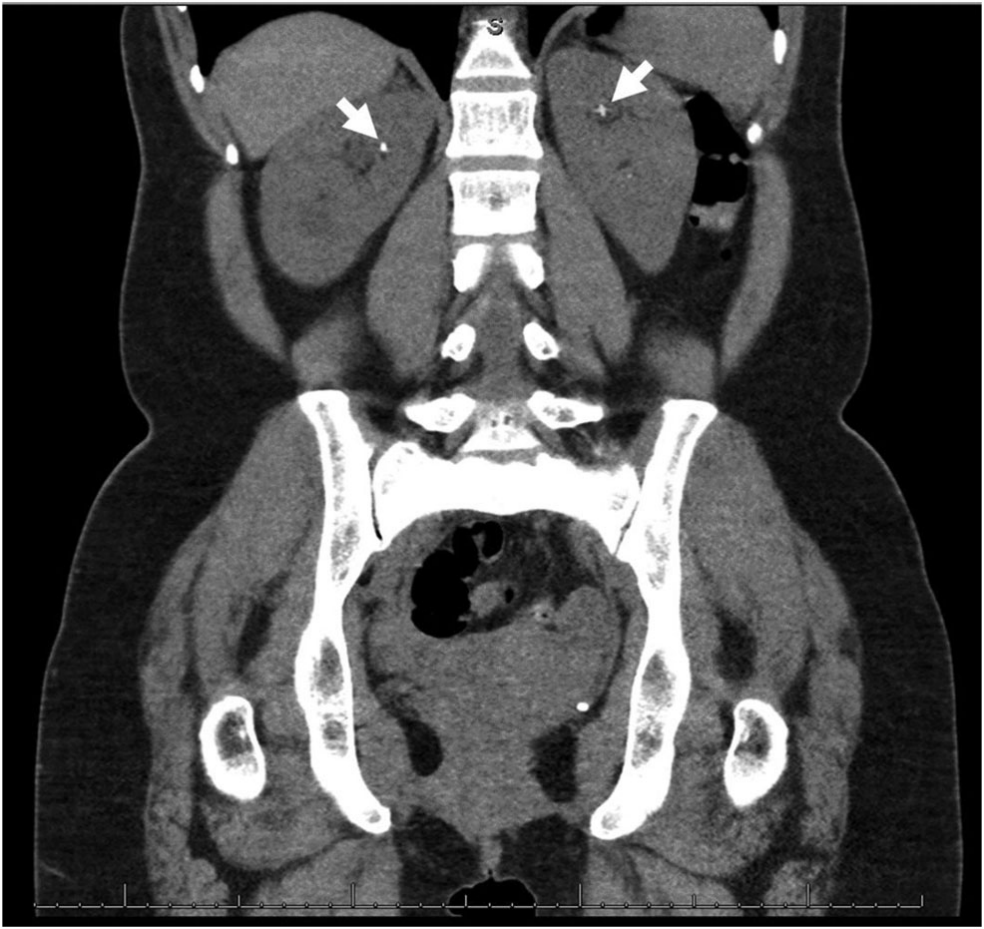
CT of kidneys, ureters and bladder (coronal view) showing bilateral renal calculi (case 2)

A 43 year-old woman presented with vitamin D insufficiency, hyperparathyroidism and normal Ca levels. Despite having vitamin D levels corrected to 78 nmol/l, her PTH levels continued to be elevated (10.2 pmol/l) while Ca levels (2.45 mmol/l, 9.8 mg/dl) remained within the reference range. Further investigations showed her CCCR was raised at 1.39% suggesting PHPT. Ultrasound scan and ^99m^Tc-sestamibi-SPECT/CT both showed a eutopic parathyroid adenoma located posterior to the inferior pole of the left thyroid lobe (Table 1). A CT scan of her kidneys showed the presence of bilateral renal calculi (Fig. 2). She had normal renal function and no evidence of osteoporosis. The patient underwent successful parathyroidectomy.

## Case 3: PHPT due to an elusive eutopic parathyroid adenoma (not identifiable by ultrasound scan and ^99m^Tc-sestamibi-SPECT/CT)

A woman in her seventies presented with hypercalcaemia (Ca = 3.00 mmol/l, 12.0 mg/dl) and hyperparathyroidism (PTH = 10.9 pmol/l), and her CCCR was elevated at 1.5% (Table 1). She had vitamin D insufficiency (69 nmol/l) with normal renal function (eGFR >60 ml/min). She had evidence of osteoporosis but without renal calculi. Both an ultrasound scan and ^99m^Tc-sestamibi-SPECT/CT could not identify either eutopic or ectopic parathyroid adenoma. She underwent neck exploratory surgery where the right superior parathyroid gland was removed, but her PTH and Ca levels failed to normalise. A four-dimensional computerised tomography (4D CT) scan was subsequently performed showing evidence of a parathyroid adenoma in the inferior pole of the left thyroid lobe. The patient underwent successful parathyroidectomy the second time with post-operative Ca (2.40 mmol/l, 9.6 mg/dl) and PTH (3.3 pmol/l) levels falling to within reference limits. It is possible that this patient had double parathyroid adenomas which were not completely excised from the initial surgical neck exploration; double adenomas occur in about 10% of patients with PHPT [15].

## Case 4: PHPT due to ectopic parathyroid adenoma

**Fig. 3:**
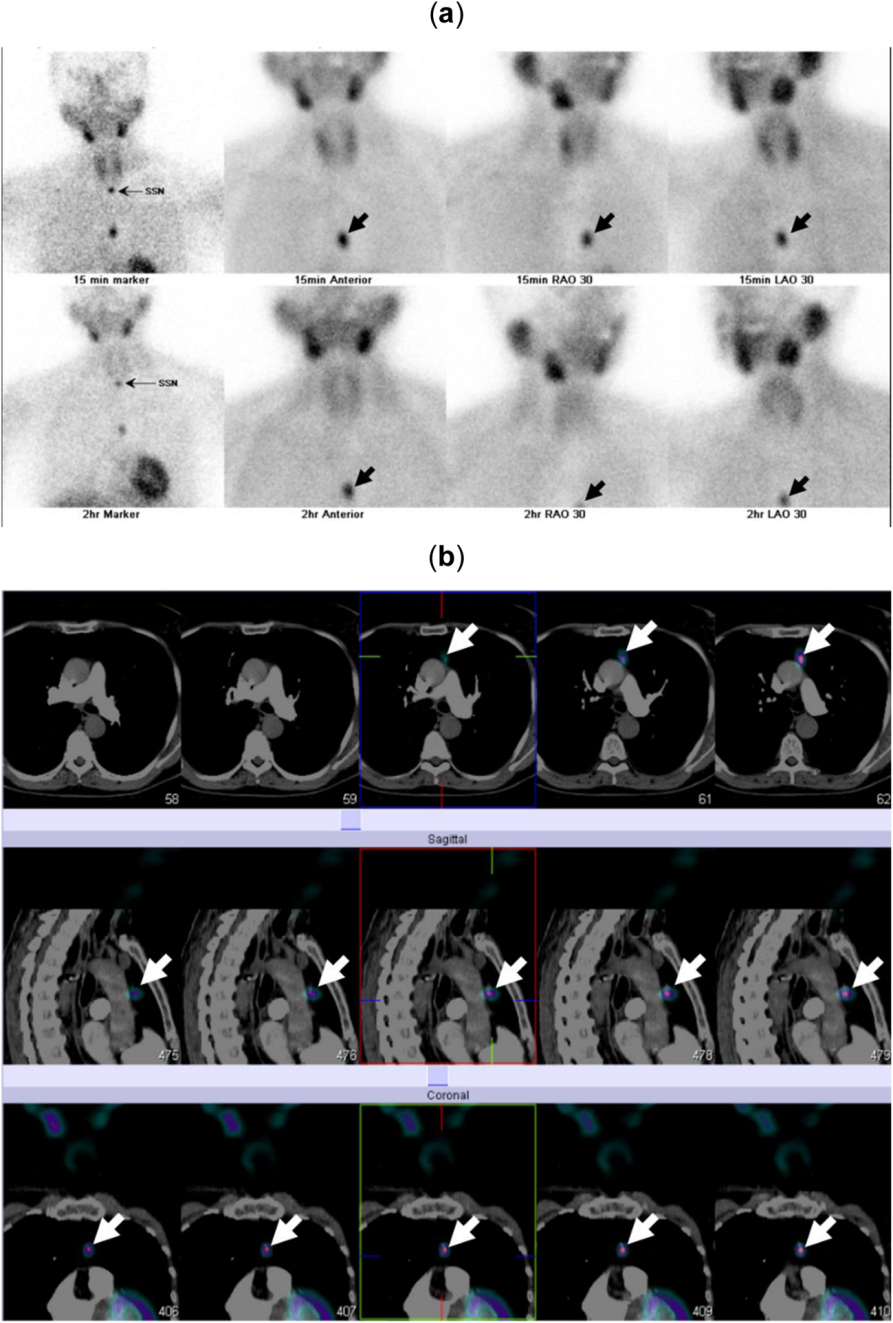
Anterior maximum intensity projection images from early (15 min) and delayed (2 h) phases of SPECT (**a**) and delayed-phase coronal, sagittal and axial fused ^99m^Tc-Sestambi-SPECT/CT images (**b**) in a woman (case 4) showing ectopic parathyroid adenoma in the anterior mediastinal fat sited just behind the sternum at level of the pulmonary trunk bifurcation

A woman in her sixties presented with hypercalcaemia (Ca = 3.23 mmol/l, 12.9 mg/dl), hyperparathyroidism (PTH = 42.1 pmol/l) and a high CCCR (2.8%). She had vitamin D insufficiency (57 nmol/l) and osteoporosis. Her renal function was normal and an ultrasound of her kidneys showed no evidence of calculi (Table 1). An ultrasound scan of her neck could not locate a eutopic parathyroid adenoma. However, ^99m^Tc-sestamibi-SPECT/CT showed an ectopic parathyroid adenoma in the anterior mediastinal fat sited behind the sternum at the level of the pulmonary trunk bifurcation. The adenoma extended to a craniocaudal extent of approximately 2 cm (Fig. 3). The patient was referred to a cardiothoracic surgeon for further evaluation including a 4D CT scan. While waiting for surgery, the patient required repeated pamidronate infusions due to persistently elevated Ca levels of over 3 mmol/l (12.0 mg/dl). Left video-assisted thoracoscopic surgery (VATS) was performed to excise this ectopic parathyroid adenoma leading to normalisation of Ca (2.46 mmol/l, 9.9 mg/dl) and PTH (6.4 pmol/l) levels.

## Case 5: Syndromic PHPT

**Fig. 4:**
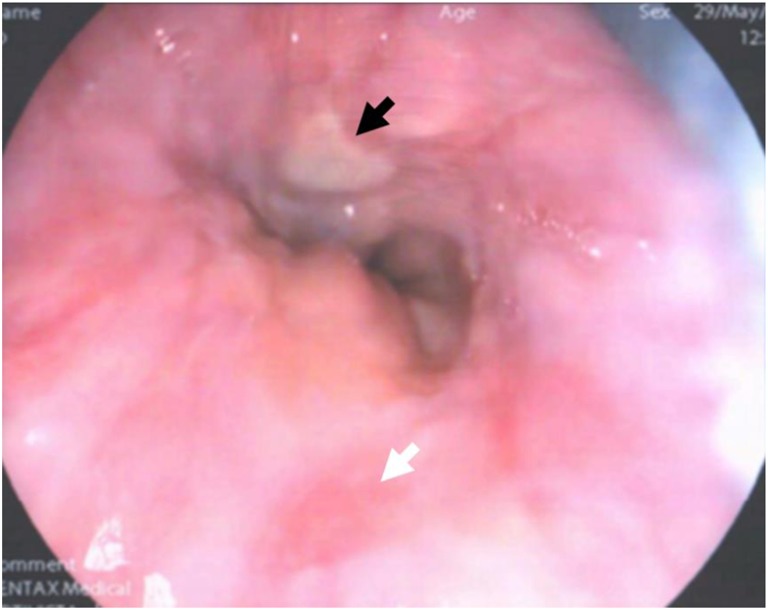
Oesophago-gastro-duodenoscopy image showing a fresh ulcer (black arrow) and healing ulcer (white arrow) in a young man presented with severe anaemia and hypercalcaemia (case 5)

**Fig. 5:**
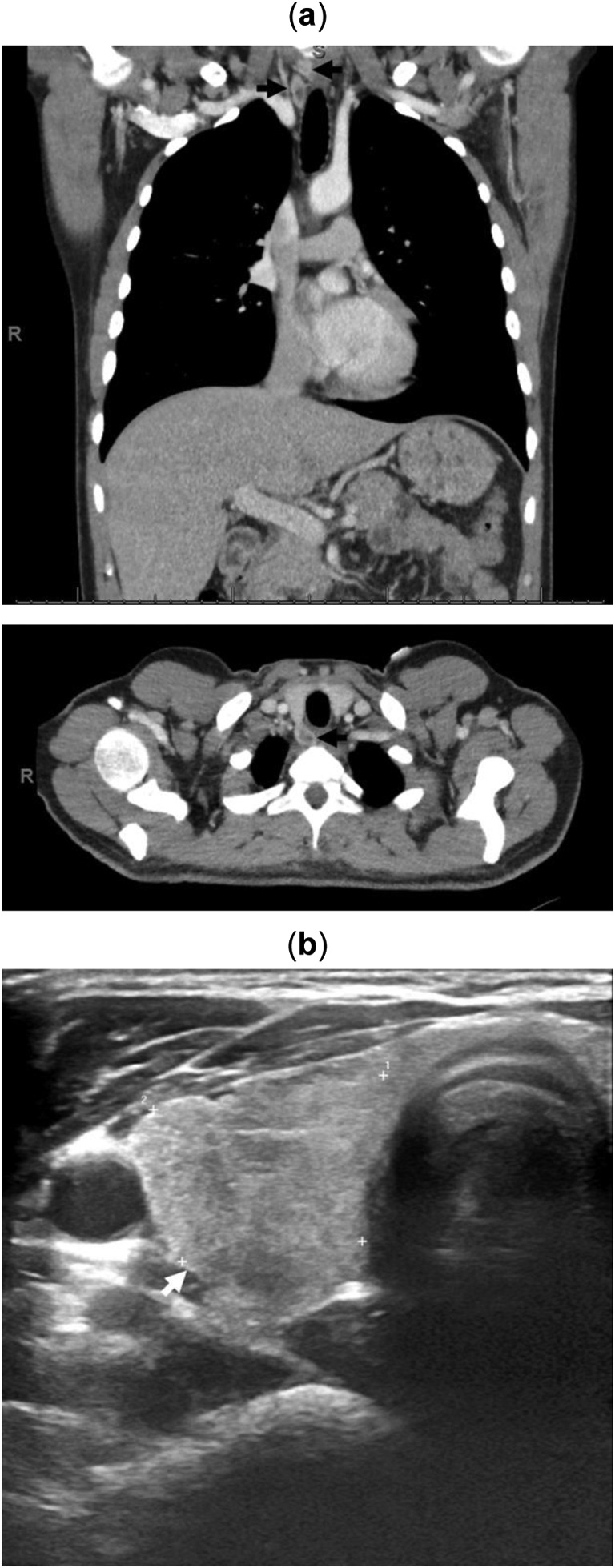
Two parathyroid adenomas identified by CT (**a**) and ultrasound scan (**b**) located posterior and inferior to the right thyroid lobe in a young man (case 5)

A 38 year-old man presented with malaena. He was admitted to hospital by a medical team where severe anaemia was discovered with Hb = 53 g/l (reference range: 130–180 g/l) and a raised urea (10.1 mmol/l; reference range: 2.5–7.8 mmol/l). He immediately received transfusion of three units of red blood cells and underwent oesophago-gastro-duodenoscopy which revealed a fresh ulcer, as well as a healing ulcer at the gastro-oesphageal junction (Fig. 4). A CT scan of his chest, abdomen and pelvis revealed small non-obstructing calculi in both kidneys, sclerotic bones and a small structure at the base of the neck, posterior to the right lobe of the thyroid. The latter was suggestive of two discrete parathyroid adenomas (Fig. 5a), which was supported by an ultrasound scan of the neck (Fig. 5b). Ca and PTH levels were elevated at 3.23 mmol/l (12.9 mg/dl) and 37.6 pmol/l respectively while there was evidence of vitamin D insufficiency (44 nmol/l). He was referred to the endocrine team for further assessment.

**Fig. 6:**
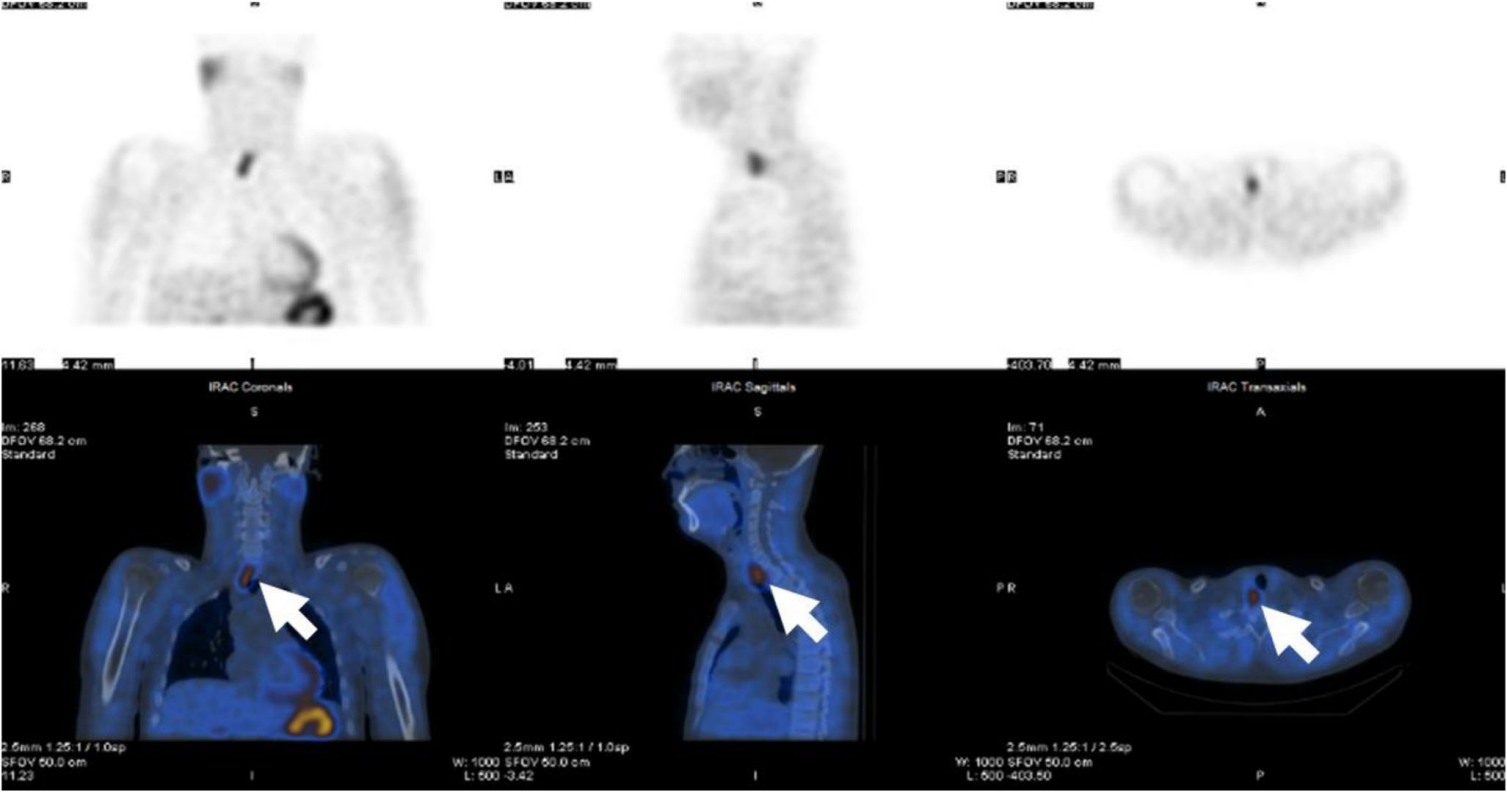
Anterior maximum intensity projection images of SPECT showing delayed washout of radiotracer (upper panel) and delayed-phase coronal, sagittal and axial fused ^99m^Tc-Sestambi-SPECT/CT images showing a parathyroid adenoma localising posterior and inferior to the right lobe of thyroid (lower panel) of a man (case 5)

Further review of this patient’s medical history showed that he had been complaining of bloating, nausea and diarrhoea for about ten years. Recently, he underwent endoscopy showing evidence of gastritis and duodenitis with a lot of erosion; there was no evidence of *Helicobacter pylori* and colonoscopy was normal. The patient was treated with lansoprazole, 30 mg per day. On direct questioning, he denied a family history of hypercalcaemia, peptic ulcers or parathyroid, pituitary or thyroid surgery. He had a brother two years his junior and he was married with two young children. He did not take regular medications, drink excessive amounts of alcohol or smoke cigarettes. Further endocrine investigations showed that his CCCR was raised at 1.02% and a DEXA scan showed normal bone mineral density. ^99m^Tc-sestamibi-SPECT/CT confirmed the CT findings and ultrasound scans showed two parathyroid adenomas lying posterior to the lower pole of the right thyroid lobe (Fig. 6). The patient underwent parathyroidectomy of the adenomas which normalised his PTH and Ca levels.

In view of his young age, screening for syndromic PTH was carried out including a number of fasting biochemical tests (Table 2). His prolactin levels were raised at 1630 mU/l. Pituitary magnetic resonance imaging (MRI) with contrast could not identify a pituitary adenoma. He had no visual field defects. Levels of IGF-1 were also raised at 32.4 nmol/l, therefore a 2-h oral glucose tolerance test was performed. Levels of gastrin were elevated to >400 pmol/l and chromogranin-A to 70 pmol/l. Fasting levels of blood glucose, insulin, remaining gut peptides including glucagon, cocaine- and amphetamine-regulated transcript (CART), somatostatin, pancreatic polypeptide and vasointestinal polypeptide, and calcitonin were all within reference limits. A 24-h urine analysis showed normal 5-hydroxyindoleacetic acid (5-HIAA) levels. After counselling, the patient agreed to undergo genetic analysis which revealed *MEN1* missense mutations (NM_130799: c.1256G > T p.(Gly419Val)) on chromosome 11 (Chr11:g.64572600C > A) causing multiple endocrine neoplasia type 1 (MEN1) syndrome. This variant has not been reported in the Genome Aggregation Database (gnomAD) [43]. The patient and his family members are currently being reviewed by specialists in the MEN1 clinic.

**Table 2:** Additional investigations for case 5 to screening for MEN syndromes

Fasting biochemical screening tests	Reference range	Results
Prolactin (mU/l)	45–175	**1630**
IGF-1 (nmol/l)	9.4–29.3	**32.4**
Glucose (mmol/l)	4.1–6.0	5.7
Insulin (pmol/l)	–	48
Gastrin (pmol/l)	0–40	**>400**
Glucagon (pmol/l)	0–50	18
CART (pmol/l)	0–129	58
Somatostatin (pmol/l)	0–150	25
Chromogranin A (pmol/l)	0–60	**72**
Chromogranin B (pmol/l)	0–150	145
Pancreatic polypeptide (pmol/l)	0–300	46
Vasointestinal polypeptide (pmol/l)	0–30	6
Calcitonin (ng/l)	0–11.8	3.2
24 h urine 5-HIAA (μmol/24 h)	0–47	37
2-h OGTT		
Baseline: GH (μg/l), glucose(mmol/l), IGF-1(nmol/l)		3.7, 4.9, 33.9
GH at 30, 60, 90 and 120 min		0.6, 0.2, 0.1, 0.1
Glucose at 30, 60, 90 and 120 min		13.2, 12.1, 11.0, 5.4
Genetic analysis		
*MEN1*		NM_130799: c.1256G > Tp.(Gly419Val)
Radiological investigations		
CT chest, abdomen and pelvis		2 × 1.5 cm solid structure posterior to the right thyroid lobe suggestive of two parathyroid adenomas Bilateral non-obstructing renal calculi (2 mm) Bones appear sclerotic Spleen, pancreas and adrenal glands appear normal
Ultrasound neck		Two parathyroid adenomas in right thyroid lobe
DEXA		Osteopenia
^99m^Tc-sestamibi-SPECT/CT		Right parathyroid adenomas
MRI pituitary with contrast		No pituitary adenoma identified
Oesophago-gastro-duodenoscopy		Ulcers at gastro-oesophageal junction
Visual field test		No VF defects

Boldface indicates value over reference range

IGF-1, insulin like growth factor-1; CART, cocaine- and amphetamine-regulated transcript; 5-HIAA, 5-hydroxyindoleacetic acid

## Case 6: Familial hypocalciuric hypercalcaemia due to *CaSR* mutation

A young woman of 43 years of age presented with hypercalcaemia (Ca 2.8 mmol/l, 11.2 mg/dl), normal PTH levels (4.4 pmol/l) and low CCCR of 0.31% (Table 1). She had normal vitamin D levels (75 nmol/l) and renal function (eGFR >60 ml/min). Collateral history revealed that both her mother (in her fifties) and maternal grandmother (in her eighties) were investigated for hypercalcaemia, but no diagnosis or treatment was offered for either.

Further investigations of this woman showed no evidence of renal calculi or osteoporosis. Genetic analysis revealed a deletion mutation on chromosome 3 (Chr3: g.122003747del) resulting in a *CASR* frameshift variant starting at codon 983 with the new reading frame ending in a termination codon 25 codons downstream. This variant affected a region which was critical for CASR function, as demonstrated by the presence of an additional pathogenic frameshift and missense variants within the affected region. This variant has been reported at a low frequency (1 in 125,707 heterozygous) in the gnomAD database [43]. This patient was diagnosed with FHH type 1 (MIM45980) and therefore did not require treatment. Based on this patient’s strong family history of hypercalcaemia and the pattern of inheritance of an autosomal dominance *CASR* gene, it is likely that both her maternal grandmother and mother carried the defected *CASR* gene which was passed on to the patient. Her offspring have a 50% risk of inheriting this variant and being predisposed to FHH. It is therefore essential that her family has comprehensive review by clinical geneticists to identify affected and unaffected family members.

## Case 7: Secondary hyperparathyroidism

A 56 year-old man with type-1 diabetes and end-stage renal disease, who has been on haemodialysis for a number of years. He has developed hyperphosphataemia and vitamin D deficiency. His Ca levels were at the lower end of the reference range (2.15 mmol/l, 8.6 mg/dl) and PTH was raised at 77.3 pmol/l. He was treated with phosphate binders and calcitriol but the hyperparathyroidism did not improve. He complained of worsening symptoms of musculoskeletal pain on minimal exertion. Treatment with the calcimimetic cinacalcet was started with an initial dose of 30 mg daily followed by dose adjustment every four weeks. Soon after treatment, the patient’s symptoms improved substantially and PTH levels were reduced to 10 pmol/l within six months of treatment.

## Discussion

The five distinct cases with hypercalcaemia and two with normocalcaemia exemplify general investigations and management of patients who presented with parathyroid gland dysfunction. Their characteristics are consistent with those reported in the published literature [18–24]. There are a number of features that distinguish PHPT from FHH (Table 3). Patients with FHH present at a younger age than those with PHPT: hypercalcaemia may be present in children with FHH before ten years of age, which is rarely observed in other forms of familial hyperparathyroidism [44]. By contrast, patients with PHPT can exhibit a number of symptoms and are at increased risk of osteoporosis and renal calculi. Our case 5 is of a young man who presented with peptic ulcers which is infrequently reported in literature [19]. Patients with PHPT often present with elevated alkaline phosphatase levels, which are reversed by vitamin D repletion [45, 46] or parathyroidectomy [47]. Patients with PHPT often have co-existing vitamin D insufficiency due to an accelerated catabolism of this hormone [22]. Repletion of vitamin D in such patients is recommended [28] which may lead to reduction of PTH levels and bone turnover while hypercalcaemia is reassuringly not exacerbated [46]. One of our cases (case 2) presented with normal Ca and high PTH levels but displayed typical features of PHPT including a raised CCCR and renal calculi, and the presence of a parathyroid adenoma identified both by ultrasound scan and ^99m^Tc-sestamibi-SPECT/CT. This condition is relatively common and is thought to progress gradually to hypercalcaemic hyperparathyroidism [48]. A study of 100 Spanish postmenopausal women identified six cases (6%) with normocalcaemic hyperparathyroidism and vitamin D >30 ng/ml (75 nmol/l) [49]. In a Canadian study [50], up to 16.7% of such cases were described, but here the level of vitamin D insufficiency was set at a lower level (<20 ng/dl, 50 nmol/l) and renal insufficiency was not excluded: therefore this figure might have been overestimated by the inclusion of some individuals with secondary hyperparathyroidism. Individuals with normocalcaemic hyperparathyroidism have similar clinical and biochemical characteristics to those with secondary hyperparathyroidism, except for the absence of vitamin D deficiency and chronic kidney disease in normocalcaemic hyperparathyroidism [48]. Secondary hyperparathyroidism occurs in 30–50% of patients treated with haemodialysis in Europe, slightly more in North America (54%), but much lower in Japan (11.5%) [51]. In these patients, hyperphosphataemia arises from a reduction in renal phosphorus clearance, resulting in an increase of fibroblast growth factor-23 (an important factor controlling phosphate metabolism), vitamin D deficiency or resistance and hypocalcaemia. All these factors stimulate parathyroid cell proliferation and consequent parathyroid hyperplasia [52]. Treatment of such secondary hyperparathyroidism is by active vitamin D3 metabolites such as alfacalcidol or calcitriol and phosphate binders. Treatment with the calcimimetic cinacalcet (30–180 mg daily) for up to 64 months for haemodialysis patients attenuates the progression of secondary hyperparathyroidism to severe unremitting (tertiary) hyperparathyroidism: relative hazard (cinacalcet versus placebo) = 0.31 (95% confidence interval = 0.26–0.37) [53], and thus reduces the need for parathyroidectomy.

**Table 3:** Clinical features differentiating PHPT from FHH

	PHPT	FHH
Age at presentation	Older	Younger
Family history	± ^†^	+++
Symptoms	++	–
Hypercalcaemia	+++	++
Hyperparathyroidism	+++	±
Calcium/creatinine clearance ratio	>1%	<1%
Vitamin D insufficiency	++	–
Raised alkaline phosphatase	++	–
Ultrasound scan	+++	–
^99m^Tc-sestamibi-SPECT/CT	+++	–
Osteopenia/osteoporosis	++	–
Renal calculi	++	–
Peptic ulcers	+	–
Curative parathyroidectomy	+++	–

Strength of association: +++ = very strong, ++ = moderate, + = mild, − = less likely

^†^Family history may be present in familial forms of PHPT

Screening for MEN syndromes is recommended [54] for patients with: PHPT before the age of 40 years; a family history of hypercalcaemia; prior unsuccessful parathyroid surgery; or hypercalcaemia identified before 25 years in a patient or relative. Case 5 in our series expressed a number of clinical features suggestive of a syndromic form of PHPT whose diagnosis was confirmed by DNA analysis. The process of tumour detection involved biochemical screening tests including gastrin, glucose, insulin, chromogranin-A, pancreatic polypeptide, glucagon, vasointestinal polypeptide, prolactin and IGF-1 to assess for gastrinoma, insulinoma, enteropancreatic and anterior pituitary tumours. Radiological examinations such as MRI or CT are helpful to detect pituitary, pancreatic, adrenal or carcinoid tumours while DNA analysis helps ascertain germline mutations including *MEN1, RET* and *CDKN1B* [29, 30].

The common eutopic parathyroid adenomas are routinely treated with parathyroidectomy while the less common ectopic parathyroid adenomas require more complex investigations and surgical procedures such as VATS. Once localised, parathyroidectomy is curative. Occasionally, both ultrasound and ^99m^Tc-sestamibi-SPECT/CT scans fail to localise the lesion and additional procedures may be necessary. More recently, 4D CT has been used in such cases [55], thus avoiding invasive procedures such as surgical neck exploration [56] or selective parathyroid venous sampling [57]. A recent study showed that among six patients where ^99m^Tc-sestamibi-SPECT/CT did not localise the lesion, surgeon-performed peri-operative ultrasound scans successfully located the parathyroid adenoma in three (50%) patients [41]. Infrequently, when parathyroid surgery is inappropriate or contra-indicated in patients with PHPT, cinacalcet (a calcimimetic agent) may be considered [58]. This may normalise hypercalcaemia in up to 80% of patients, but it does not prevent bone loss or kidney problems [59]. Calcimimetics regulate Ca homeostasis by increasing the sensitivity of the CaSR to circulating Ca^2+^, thus reducing PTH levels and consequently decreasing tubular resorption of Ca^2+^.

On the other hand, hypercalcaemia of the much rarer autosomal-dominant FHH does not require treatment [28]. For syndromic forms of PHPT or FIHPT, once DNA sequencing confirms the presence of gene mutations, assessment of kin is important to provide appropriate management for family members (50% of offspring) who may harbour familial germline mutation. It is also important to reassure unaffected members to allay any anxiety of inheriting this condition and reducing, as well as minimising healthcare costs in avoiding unnecessary investigations and treatment.

## Clinical perspectives

Traditionally, CCCR requires assessment of serum Ca and creatinine paired with a 24-h urinary Ca and creatinine, which is not only time-consuming, costly and inconvenient, but also can result in incomplete collections that generate misleading results. We suggest that a small (5 ml) fasting urine sample, used to estimate fractional excretion of Ca (FE_Ca_), can provide a reliable and practical method for the differential diagnosis of hypercalcaemia. The costs and ease of screening tests would be reduced substantially, with an expected improvement in compliance of sample collection. The method could potentially be used more widely, including in a primary-care setting.

A study was conducted to determine the relationship between CCCR and FE_Ca_ and assess identification of PHPT in 13 men and 38 women, age 20–93 years. Fifteen were undergoing routine investigations for PTH-related hypercalcaemia at The Royal Free Hospital, University College London: the remainder were healthy participants who served as a control set [39, 40]. It found that in all patients with PHPT, CCCR calculated either from 24-h urine or from a 5 ml sample was greater than the cut-off of 1% for discriminating PHPT from FHH. Thus, using the estimated CCCR did not result in false negatives while the positive prediction and sensitivity values were 100% [39] with good test-retest repeatability [40].

To evaluate the clinical application of this simplified method, a 5 ml aliquot of fasting urine and a 24-h urine sample were collected for our patient with FHH (case 6). We found identical CCCR values of 0.31% measured from either a 5 ml aliquot or a 24-h urine collection in this patient, which is well below 1% threshold for diagnosis of FHH. However, a cross-validation study of patients with this rare condition, as well as those with PHPT, is required to confirm the applicability of using a 5 ml aliquot for assessing CCCR in routine clinical practice.
